# Low-temperature enhances production of severe fever with thrombocytopenia syndrome virus virus-like particles

**DOI:** 10.1007/s00253-025-13436-y

**Published:** 2025-03-03

**Authors:** Isabelle Loop, Yuan-Dun Ke, Wei-June Chen, Kun-Hsien Tsai, Wei-Li Hsu, Yi-Chin Fan

**Affiliations:** 1 Institute of Epidemiology and Preventive Medicine, College of Public Health, National Taiwan University, Taipei, Taiwan; 2 Department of Public Health and Parasitology, Chang Gung University, Taoyuan, Taiwan; 3 Institute of Environmental and Occupational Health Sciences, College of Public Health, National Taiwan University, Taipei, Taiwan; 4 Global Health Program, College of Public Health, National Taiwan University, Taipei, Taiwan; 5 Graduate Institute of Microbiology and Public Health, College of Veterinary Medicine, National Chung Hsing University, Taichung, Taiwan; 6 Master of Public Health Program, College of Public Health, National Taiwan University, Taipei, Taiwan

**Keywords:** Severe fever with thrombocytopenia syndrome virus, Genotype, Glycoprotein, Virus-like particle, Low-temperature cultivation, Mammalian cells

## Abstract

**Abstract:**

Tick-borne severe fever with thrombocytopenia syndrome (SFTS) is an emerging zoonotic disease caused by the SFTS virus (SFTSV). Serological assays based on the nucleocapsid protein and partial glycoprotein of this virus have been used for detecting SFTSV infections in humans and animals. However, whether the complete SFTSV glycoprotein (Gn/Gc) can induce the assembly of virus-like particles (VLPs) which can be used for serological surveillance and vaccine production remains unclear. In this study, we successfully expressed and secreted SFTSV Gn/Gc antigens by using a single plasmid encoding the complete glycoprotein sequence of the dominant genotype B virus. HEK293T and COS-1 cells were transfected with the aforementioned plasmid; cultivating these cells at 32 °C, instead of 37 °C, led to 4.0- and 3.3-fold higher antigen recovery, respectively. The secreted Gn/Gc antigens at 32 °C retained epitopes resembling those of the virion; these epitopes were recognized by a SFTS human–derived monoclonal antibody. Sucrose density gradient centrifugation, followed by transmission electron microscopy, confirmed the formation of VLPs with a diameter of approximately 100 nm. Overall, our findings highlight the potential of SFTSV VLPs for serological surveillance and vaccine development.

**Key points:**

• *Cultivating transfected cells at 32 °C boosts SFTSV glycoprotein production.*

• *Complete SFTSV glycoprotein expression facilitates virus-like particle assembly.*

• *The assembly does not require any other viral proteins or RNA.*

**Supplementary Information:**

The online version contains supplementary material available at 10.1007/s00253-025-13436-y.

## Introduction

Severe fever with thrombocytopenia syndrome (SFTS) virus (SFTSV) is a tick-borne pathogen that poses a major threat to human health. SFTSV emerged in China in 2009 and has since been reported across East Asia, including countries such as Japan, South Korea, Vietnam, and Taiwan (Casel et al. 2021). This virus causes SFTS, which is characterized by fever, thrombocytopenia, leukocytopenia, fatigue, and gastrointestinal symptoms (Gai et al. 2012). SFTS exhibits case-fatality rates of 1.6 to 29.8% in China, 23.3% in South Korea, and 27% in Japan (Li et al. 2022; Yun et al. 2020).

In 2018, the World Health Organization and National Institute of Allergy and Infectious Diseases designated SFTSV as a priority pathogen. However, despite extensive efforts to develop SFTSV therapeutics and vaccines, none have yet to be approved for clinical use (Kim et al. 2024).

Although the virus has been classified as *Bandavirus dabieense* under the *Phenuiviridae* family, it is commonly known as SFTSV (Schoch et al. 2020). The virus genome comprises three negative-sense RNA strands: large (L), medium (M), and small (S) segments. The L segment encodes an RNA-dependent RNA polymerase. The M segment encodes a polyprotein precursor that is processed into two surface glycoproteins called Gn and Gc. The S segment encodes nucleoprotein (NP) and nonstructural (NSs) proteins in an ambisense orientation (Wu et al. 2017; Yu et al. 2011). Phylogenetic analysis of the S segment has classified SFTSV strains into genotypes A through F. Geographic variation has been noted in the dominant strains of SFTSV. For example, genotype F is prevalent in China, whereas genotype B (GB) is more prevalent in South Korea and Japan (Yun et al. 2020). In Taiwan, GB has been detected in humans, whereas genotype C has been detected in animals (Lin et al. 2020; Peng et al. 2020). Genotype-specific pathogenicity may explain the lower case-fatality rate in China than in South Korea and Japan (Yun et al. 2020).

Ticks are the primary vector for SFTSV, and ruminants facilitate maintenance of the virus in nature and its transmission to humans (Kuan et al. 2023; Zhuang et al. 2018). In animals, viral RNA–based diagnosis of SFTSV infection is challenging because of the short duration of viremia, rare presence of clinical symptoms, and low prevalence of viral RNA in domestic and wild animals (Huang et al. 2019; Jiao et al. 2015; Kang et al. 2019; Niu et al. 2013). Thus, serological methods are used for detecting SFTSV infection in animal and asymptomatic infection in human (Huang et al. 2017). The virus neutralization test is considered to be the gold standard for diagnosing SFTSV (Jiao et al. 2012). However, this test is time-consuming, is costly, and requires a high biosafety-level (BSL) laboratory capable of handling live viruses. The enzyme-linked immunosorbent assays (ELISAs) is a rapid, economical, and reliable method. Previously developed SFTSV ELISAs include a competitive ELISA detecting total antibodies against a purified recombinant SFTSV NP and an indirect ELISA targeting the NP (Jiao et al. 2012; Yu et al. 2015). The aforementioned studies focused on the NP because it is a well-known immunogenic protein and is encoded on the S segment, the most conserved segment of the SFTSV genome (Shi et al. 2017; Yu et al. 2015).

The M segment is less conserved than is the S segment. Nonetheless, the diversity of the M segment can aid in genotyping and surveillance. In human patients, SFTSV glycoproteins induce long-lasting antibodies (Chung et al. 2022). The monoclonal antibody MAb4-5, which was isolated from a human SFTS case, neutralizes SFTSV by targeting Gn (Guo et al. 2013). The assays have been developed using the ectodomains of Gn or Gc, but none of these assays involve expressing the complete glycoprotein for detecting SFTSV antibodies (Shimojima et al. 2022).

The Gn/Gc facilitate the formation of self-assembling protein structures called virus-like particles (VLPs), as observed in related viruses such as Uukuniemi virus and Rift Valley fever virus (Overby et al. 2006; Piper et al. 2011; Zeltins 2013). VLPs retain conformational epitopes but lack infectious genetic material; thus, they can be safely produced in low-BSL laboratories (Noad and Roy 2003). The closest system for SFTSV researches to date is a reverse genetics platform comprising an M-segment-based minigenome, a NP-expressing clone, and a polymerase-expressing clone (Brennan et al. 2015). We aim to develop an alternative, noninfectious, and single-plasmid platform capable of mimicking the native virus. In this study, we investigated whether VLPs can be produced by transfecting host cells with a single plasmid carrying the complete glycoprotein sequence of SFTSV GB. We further optimized the cell culture conditions to enhance the production of VLP.

## Materials and methods

### Plasmid construction

The complete glycoprotein sequence of the SFTSV GB strain TP1910a (GenBank accession number: MN830174) was codon-optimized for efficient protein expression in human-derived cells (Fig. S1). The sequence was amplified using the GoTaq Green Master Mix (Promega, Wisconsin, USA) with 5’-AAGGTACCGCCGCCGCCATGA-3’ and 5’-ATCTCGAGTCAGGCCAGCTTGGCTCT-3’ primers (OligoAnalyzer™ Tool; Integrated DNA Technologies, Coralville, IA, USA). The resultant PCR product was inserted into the pcDNA3.1-Hygro (CMV-IE promoter) and pH (CAG promoter) vectors. The inserts and vectors were digested using *Kpn*I and *Xho*I restriction enzymes and ligated using T4 DNA ligase (Thermo Fisher Scientific, Waltham, MA, USA). SFTSV plasmids were selected from transformed *Escherichia coli* DH5α cells, and the inserts were confirmed through sequencing (Table 1).

**Table 1:** SFTSV Gn/Gc antigens-expressing plasmids used in this study

Plasmids	Promoters^1^	Gn/Gc	Codon optimization
pcDNA3.1-Hygro-SFTSV GB	CMV-IE	Genotype B	+
pH-SFTSV GB	CAG	Genotype B	+

^1^*CMV-IE* Cytomegalovirus-immediate early, *CAG* CMV early enhancer element, the first exon and the first intron of chicken beta-actin gene, and the splice acceptor of the rabbit beta-globin

### Cell culture

HEK293T cells (provided by Dr. Wei-Li Hsu, National Chung Hsing University, Taiwan) and COS-1 cells (Bioresource Collection and Research Center, Hsinchu City, Taiwan) were cultured in Dulbecco’s Modified Eagle Medium (DMEM) supplemented with 10% fetal bovine serum (FBS), 1 × minimal essential medium (MEM) non-essential amino acids solution and penicillin–streptomycin solution (Thermo Fisher Scientific).

### Transfection

HEK293T and COS-1 cells were transfected with plasmids by using Lipofectamine 3000 (Invitrogen, Waltham, MA, USA), following the manufacturer’s instructions. In brief, 1.5 mL of DMEM was added to each well of a 12-well plate, and HEK293T or COS-1 cells were seeded in each well (density: 3.0 × 10^5^ per well). A transfection mixture comprising 1.5 µL of Lipofectamine 3000, 1 µg of SFTSV plasmid DNA, and 2 µL of P3000 reagent was prepared in Opti-MEM (Thermo Fisher Scientific) and incubated at room temperature for 15 min. Then, the reagent mixture was added to each well, and the cells were incubated for 5 h at 37 °C under 5% CO2. After this incubation, the Opti-MEM was replaced with 1 mL of DMEM supplemented with 10% FBS. The cells were incubated for an additional 1 or 2 days before fixation for an immunofluorescence assay (IFA) or collection of cell lysate and supernatant, respectively.

### IFA

HEK293T and COS-1 cells were fixed in 4% formaldehyde-PBS for 30 min at room temperature. Fixed cells were permeabilized with 0.5% triton X-100-PBS for 10 min at room temperature and then blocked with 5% milk-1X PBS with Tween 20 (PBST) for 60 min. Subsequently, the cells were incubated overnight at 4 °C with primary antibody—rabbit anti-SFTSV antibodies against either Gn (Rabbit SFTS Virus HB29 Polyclonal Antibody [MBS150145]; MyBioSource, San Diego, CA, USA] or Gc peptides (SFTSV HB29 polyclonal antibody [PAB27173]; Abnova, Taipei, Taiwan]. The next day, the primary antibodies were removed, and the cells were washed with 1 × PBST. Afterward, they were stained with Alexa Fluor 488-conjugated goat anti-rabbit IgG (dilution: 1:1000; Thermo Fisher Scientific) for 1 h at room temperature. Next, the cells were washed again with 1 × PBST and then stained with DAPI (dilution: 1:5000; Thermo Fisher Scientific) for 7 min. After this staining, cells were again washed with 1 × PBST and then visualized under an immunofluorescence microscope (IX71; Olympus, Tokyo, Japan).

### Electroporation

HEK293T and COS-1 cells were centrifuged for 5 min at 4 °C, washed with ice-cold 1 × PBS, and centrifuged again. Then, the cells were suspended in ice-cold 1X PBS such that their concentration was 2.0 × 10^7^ cells/mL, and 0.5 mL of this suspension was transferred to a reaction tube. SFTSV Gn/Gc plasmids (30 μg) were added to this tube, and the mixture was transferred to an electroporation cuvette (4 mm). A 30-ms pulse with voltage 250 V was applied to the cuvette. Electroporated cells were transferred to T175 flasks containing DMEM supplemented with 10% FBS. The following day, the medium was replaced with SFM4Megavir (SFM) (Hyclone Laboratories, Logan, UT, USA), and the cells were incubated for 6 days at 37 °C, 32 °C, or 28 °C under 5% CO2.

### Supernatant and cell lysate collection and concentration

After replacing with SFM and incubating, the supernatant was collected and centrifuged for 10 min at 4 °C. Cellular debris was discarded and the supernatant was stored at − 80 °C. Later, it was filtered through a 0.45-μm filter cup and concentrated using the Amicon Ultra-15 Centrifugal Filter Device for proteins (cutoff: 30 kDa). This process yielded an 80-fold concentrated antigen, which was stored at − 80 °C.

To collect cell lysates, HEK293T and COS-1 cells cultured in a 12-well plate were washed with ice-cold PBS and lysed with ice-cold RIPA buffer (Thermo Fisher Scientific) containing proteinase inhibitors cocktail (Complete Protease Inhibitor Cocktail Tablets; Merck Millipore, Burlington, MA, USA). The lysed cells were transferred to a centrifuge tube and vortexed intermittently (every 5 min) for 30 min. The lysate was centrifuged for 20 min at 4 °C, and the supernatant was stored at − 80 °C.

### Sucrose density gradient centrifugation

For sucrose density gradient centrifugation, 20 to 60% sucrose solutions were prepared. The density gradient was prepared by layering 2 mL of each sucrose solution, from lowest to highest sucrose percentage, into sterilized ultracentrifuge tubes (Beckman Coulter, 344,059). The tubes were stored overnight at 4 °C. The next day, the concentrated antigen was layered on top of the gradient. The tubes were placed in rotor buckets (SW 41 Swinging-Bucket Rotor; Beckman Coulter, Brea, CA, USA) and centrifuged at 107,000 × *g* for 12–16 h at 4 °C, with slow acceleration and deceleration. After this centrifugation, 500-μL fractions were collected from the top of the gradient and stored at − 80 °C.

### Western blotting

A 10% resolving gel and a 5% stacking gel were prepared for SDS-PAGE. Each sample comprised 8 μL of protein and 2-μL sample buffer (1.0 M Tris–HCl [pH 6.8], SDS, glycerol, bromophenol blue, and deionized water) with or without β-mercaptoethanol. The samples were heated at 99 °C for 5 min. The running buffer was 1X Tris–glycine (pH 8.3). Electrophoresis was conducted at 80 V for 30 min and then at 120 V for 60 min. The resultant protein bands were transferred to PVDF through incubation in transfer buffer for 1.5–2.0 h at 250 mA. The membranes were blocked with 5% milk–1 × PBST and incubated overnight with primary antibodies (diluted in 5% milk–1 × PBST). Then, the membranes were washed with 1 × PBST and incubated with secondary antibodies (diluted 1:5000 in 5% milk–1 × PBST) for 1 h at room temperature. Subsequently, membranes were washed with 1 × PBST, incubated with Western Lightning Reagent Plus (PerkinElmer, Hopkinton, MA, USA), and analyzed using the UVP ChemStudio Plus Touch imaging system (Analytik Jena US, Upland, CA, USA). The intensity of Gn or Gc signals was quantified using ImageJ (version 1.54h; National Institutes of Health).

### Antigen-capture ELISA

For ELISA, 50 μL of human anti-SFTSV Gn monoclonal antibodies (MAb4-5; Creative Biolabs, New York, USA) diluted in coating buffer was added to each well of a 96-well plate and incubated for 1 h at 37 °C. The plates were blocked with 100 μL of StartingBlock (Thermo Fisher Scientific) for 5 min. Next, 50 μL of SFTSV GB Gn/Gc antigens (diluted in PBS) was added to each well and incubated overnight at 4 °C. Subsequently, the plate was washed five times with 1X PBST and incubated for 1 h at 37 °C with 50 μL of rabbit anti-SFTSV GB Gn/Gc sera (diluted 1:100 in 5% milk –1 × PBST; produced by immunizing a rabbit with pcDNA3.1-SFTSV GB; Yao-Hong Biotechnology Inc., Taiwan). After this incubation, the plate was washed five times and then incubated with HRP-conjugated goat anti-rabbit IgG antibodies (dilution: 1:8000) for 1 h at 37 °C. Afterward, the plate was washed 10 times. Then, 100 μL of TMB was added to each well and incubated (covered) for 10 min at room temperature. The reaction was stopped by adding 50 μL of 2N H_2_SO_4_. Absorbance was measured at 450/630 nm by using a microplate reader.

### Transmission electron microscopy

For transmission electron microscopy (TEM), 200-mesh carbon-coated grids were first subjected to hydrophilic treatment. Then, 4 μL of each purified antigens was added to each grid. Excess antigen solution was removed using filter paper. The grids were washed by adding 4 μL of DDW, incubating for 30 s, and removing excess water using filter paper. Afterward, the grids were stained with 2% uranyl acetate (4 μL) for 60 s in a dark room. Excess uranyl acetate was removed using filter paper. Subsequently, the grids were illuminated under a halogen lamp for 5 min and placed overnight in a humidity-controlled storage box. TEM images were captured the next day by using JEOL-JEM 1230 (JEOL, Tokyo, Japan).

### Statistical analysis

Intergroup comparisons were performed using the Kruskal–Wallis test followed by the post hoc Dunn test. Data were analyzed using GraphPad Prism (version 8.0.2; GraphPad Software, Boston, MA). Statistical significance was set at *p* < 0.05.

## Results

### pcDNA3.1-SFTSV GB plasmid exhibits high intracellular and extracellular levels of protein expression

**Fig. 1:**
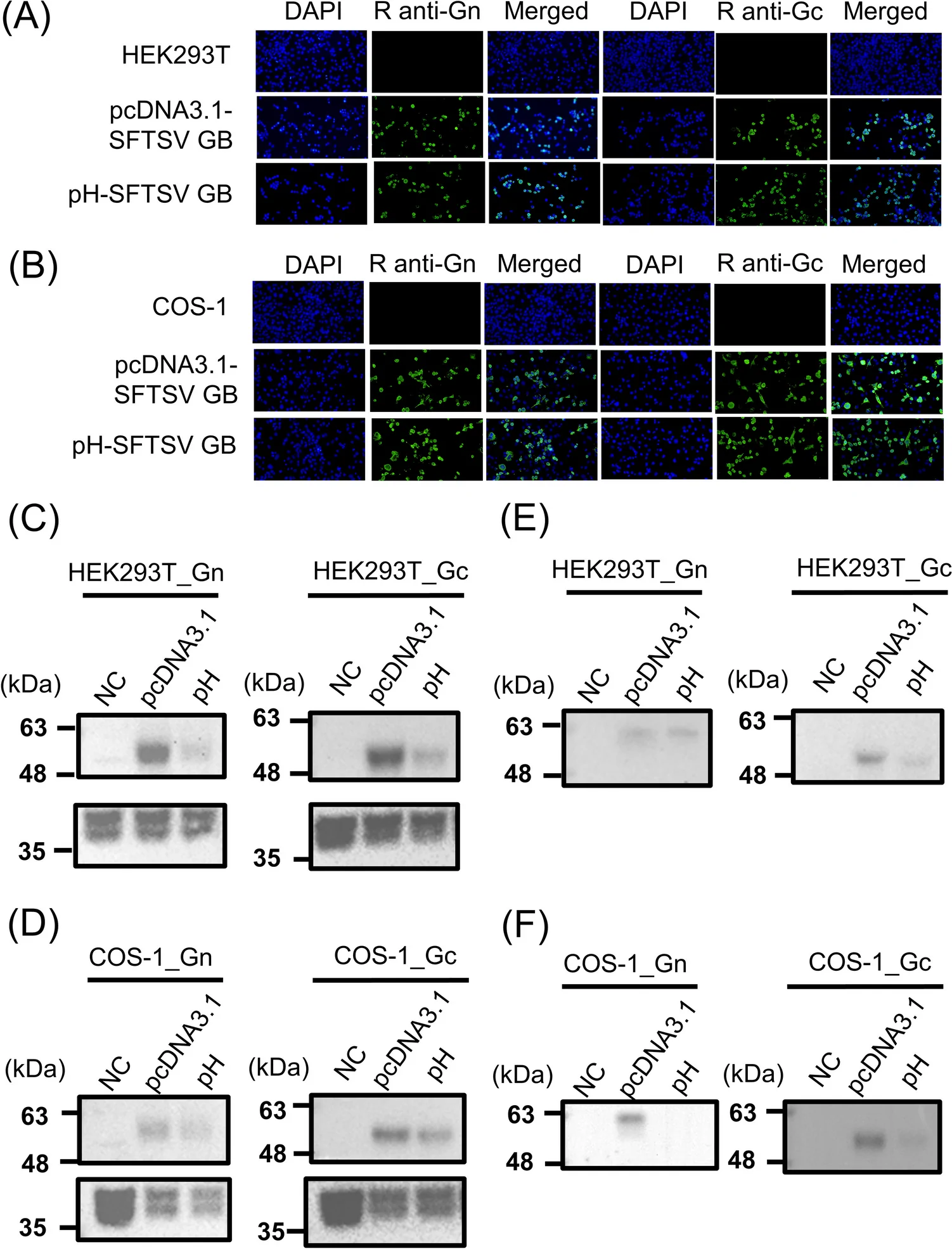
Intracellular and extracellular expression of SFTSV Gn/Gc antigens. HEK293T and COS-1 cells were transfected with pcDNA3.1-SFTSV GB or pH-SFTSV GB plasmids; both plasmids encoded a complete glycoprotein (Gn/Gc). Transfected HEK293T cells (**A**) and COS-1 cells (**B**) were stained with rabbit anti-SFTSV Gn or Gc polyclonal antibodies and analyzed through immunofluorescence assay. The nuclei were stained with DAPI. Intracellular (**C** and **D**) and extracellular (**E** and **F**) protein expression was detected through Western blotting performed using rabbit anti-SFTSV Gn (HEK293T_Gn or COS-1_Gn) or Gc (HEK293T_Gc or COS-1_Gc) polyclonal antibodies. Intracellular β-actin was stained with mouse anti-β-actin monoclonal antibodies. NC: negative control cells or cell supernatants collected from nontransfected HEK293T or COS-1cells; pcDNA3.1: pcDNA3.1-SFTSV GB; pH: pH-SFTSV GB. The amount of protein loaded and the original results of Western blotting were presented in Fig. S2. All experiments were performed in duplicated

The aforementioned case of SFTS in a human patient in Taiwan was attributed to SFTSV GB, the predominant strain circulating among animals and humans in South Korea and Japan (Peng et al.^ 2020^; Yun et al. 2020). Thus, we constructed two plasmids: pcDNA3.1-SFTSV GB and pH-SFTSV GB; these plasmids use the CMV-IE or CAG promoter, respectively, and encode the codon-optimized glycoprotein (Gn/Gc) of the GB strain (Table 1). The plasmids were transfected into HEK293T and COS-1 cells, and the intracellular and extracellular protein expression levels of these cells were compared. IFA revealed positive signals for SFTSV Gn and Gc antigens in both HEK293T and COS-1 cells transfected with the plasmids (Fig. 1A and B). However, cells without the plasmids exhibited no positive signals for the antigens. Western blotting confirmed the glycoprotein was processed into Gn and Gc antigens (Fig. 1C and D), with the expected bands at 57 and 51 kDa for Gn and Gc, respectively. For both antigens, cells transfected with pcDNA3.1-SFTSV GB exhibited stronger bands than did those transfected with pH-SFTSV GB. Therefore, pcDNA3.1-SFTSV GB induces relatively high intracellular levels of Gn and Gc expression.

Assembled SFTSV virions are enclosed within Golgi vesicles, transported to the cell surface, and then released from the cell (Lokupathirage et al. 2021). We investigated whether the complete SFTSV glycoproteins could be secreted as VLPs without any other viral proteins or RNA being required. Analyses of supernatants collected from transfected HEK293T and COS-1 cells revealed detectable Gn and Gc antigens (Fig. 1E and F). The pcDNA3.1-SFTSV GB plasmid outperformed the pH-SFTSV GB plasmid in inducing both intracellular and extracellular expression of Gn and Gc antigens. Notably, the levels of these antigens were lower in the supernatant compared than in the cell lysate (Fig. 1C and D); this result prompted exploration of various incubation temperatures to identify which temperature enhances Gn and Gc antigen secretion.

### Low-temperature cultivation enhances the secretion of SFTSV Gn/Gc antigens

**Fig. 2:**
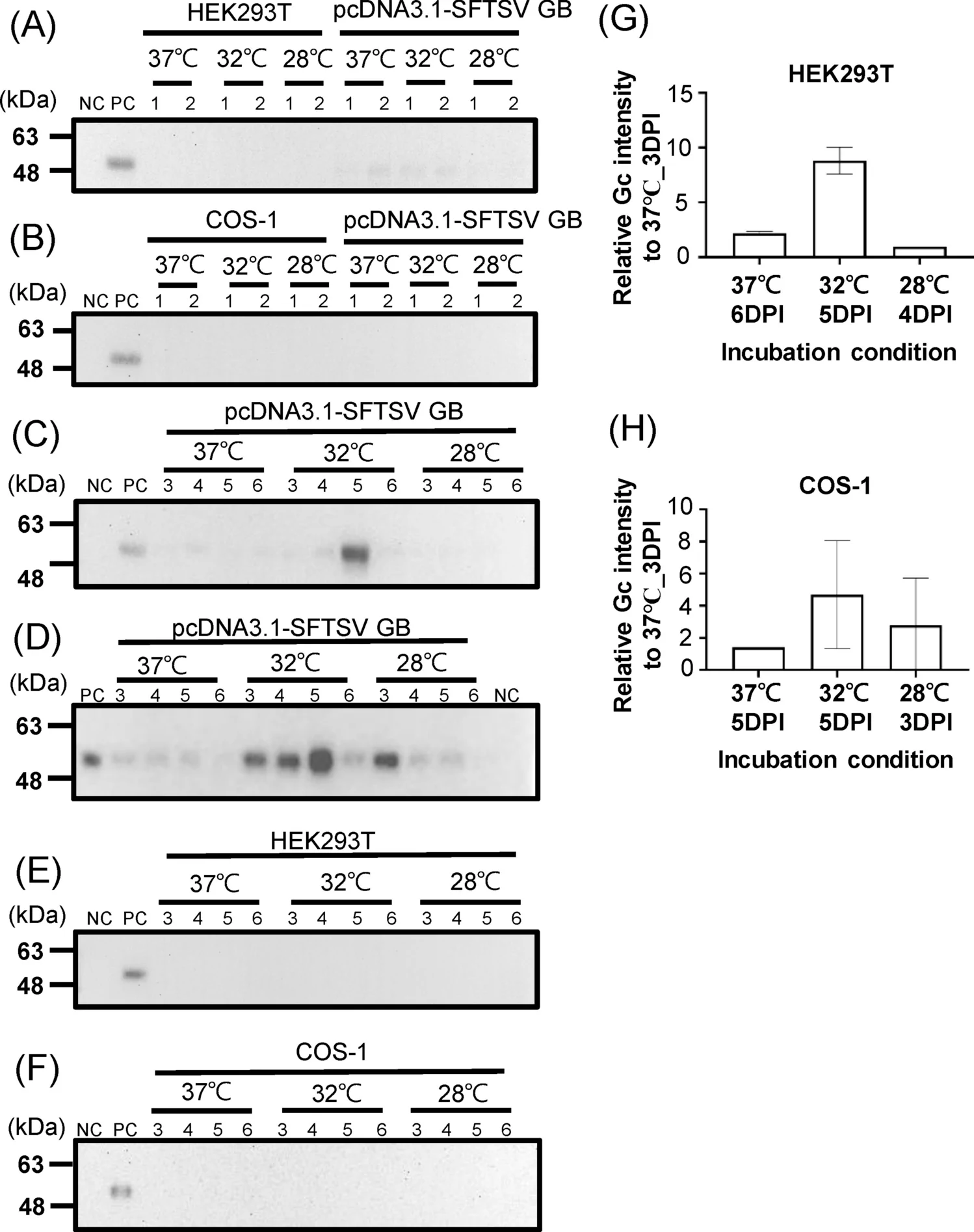
Secretion of SFTSV Gc antigens from transfected cells cultivated at different temperatures. HEK293T and COS-1 cells were transfected with pcDNA3.1-SFTSV GB and incubated at 37 ℃, 32 ℃, or 28 ℃ for 6 days. **A**–**F** Gc secretion from transfected (pcDNA3.1-SFTSV GB) and nontransfected (HEK293T or COS-1) cells at 1–6 DPI (indicated as 1, 2, 3, 4, 5, and 6). NC: negative control—supernatant collected from nontransfected HEK293T or COS-1 cells; PC: positive control—concentrated Gn/Gc supernatant. Level of Gc secretion from transfected (**G**) HEK293T or (**H**) COS-1 cells under the incubation condition with the highest intensity for each temperature, plotted against the intensity of the secreted Gc at 3 DPI at 37 ℃. The amount of protein loaded and the original results of western blotting are presented in Fig. S3. All experiments were performed in duplicate. Data are presented in terms of mean ± a standard deviation values

Studies have demonstrated that reducing the incubation temperature from 37 to 28 ℃ or 32 ℃ increased the yield of flavivirus VLPs and severe acute respiratory syndrome coronavirus 2 spike proteins (Castro et al. 2021; Fan et al. 2024). Thus, we investigated whether a reduction in incubation temperature could enhance the secretion of SFTSV Gn and Gc antigens. HEK293T and COS-1 cells transfected with the pcDNA3.1-SFTSV GB plasmid were incubated at 37 °C, 32 °C, and 28 °C. The level of Gc proteins in the supernatant was measured daily for 6 days postincubation (DPI). In HEK293T cells, faint Gc bands were detectable in the supernatants of transfected with pcDNA3.1 SFTSV-GB plasmids at 1 DPI and 2 DPI (Fig. 2A). However, no detectable Gc was observed in COS-1 cells at these time points (Fig. 2B). In HEK293T cells, expression levels peaked at 5DPI for 32 °C and were then much lower at 6 DPI (Fig. 2C). Expression of Gc was low under other incubation conditions. In COS-1 cells, the expression of Gc upregulated at 3, 4, and 5 DPI when the temperature was 32 °C (Fig. 2D). It peaked at 5 DPI and was nearly undetectable 6 DPI. Notably, when the temperature was 28 °C, a strong Gc band was visible at 3 DPI in COS-1 cells. No detectable Gc band was observed at 3, 4, and 5 DPI in both cells without plasmid transfection (Fig. 2E and F).

We quantified the highest yield of Gc antigens for each temperature relative to the band intensity at 3 DPI for 37 ℃ (Fig. 2G and H). In HEK293T cells, the highest levels of Gc secretion were detected at 6 DPI for 37 °C, 5 DPI for 32 °C, and 4 DPI for 28 °C, with the relative intensities being 2.19, 8.82, and 0.97, respectively (Fig. 2G) Statistical comparisons of incubation outcomes revealed no significant differences between 37 and 32 ℃ (*p* = 0.855), 37 ℃ and 28 ℃ (*p* = 0.855), or 32 ℃ and 28℃ (*p* = 0.097). In COS-1 cells, the highest levels of Gc secretion were discovered at 5 DPI for 37 °C, 5 DPI for 32 °C, and 3 DPI for 28 °C, with the relative intensities being 1.41, 4.67, and 2.78, respectively (Fig. 2H). Statistical comparisons of incubation outcomes revealed no significant differences between 37 and 32 ℃ (*p* = 0.544), 37 ℃ and 28 ℃ (*p* > 0.99), or 32 ℃ and 28 ℃ (*p* = 0.855). Notably, incubation at 32 °C resulted in 4.0- and 3.3-fold higher Gc yield in HEK293T and COS-1 cells, respectively, compared with the yield at 37 °C. These findings indicate that incubation at 32 °C for 5 days are the optimal conditions for producing SFTSV Gc antigens.

### MAb4-5 recognizes the secreted Gn/Gc antigens

**Fig. 3:**
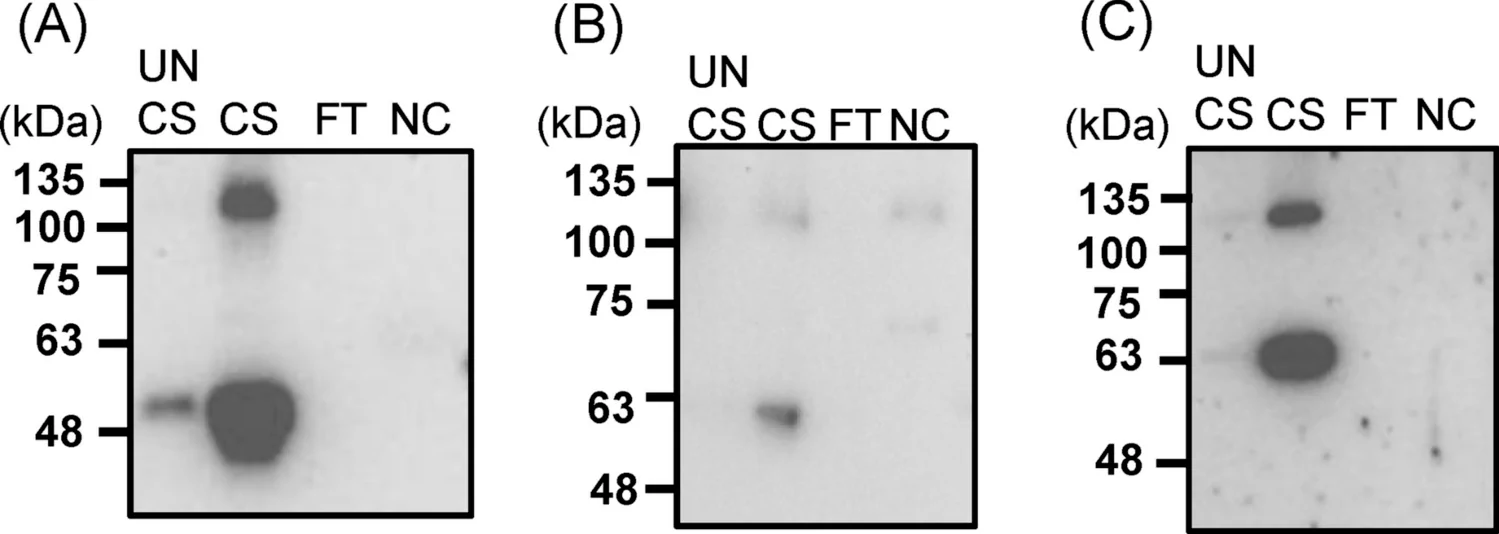
Levels of SFTSV Gn/Gc antigens secreted from transfected COS-1 cells cultivated at 32 ℃. COS-1 cells were transfected with the pcDNA3.1-SFTSV GB plasmid and incubated at 32 ℃. The supernatant collected from these cells at 5 days after incubation was concentrated using a cellulose membrane with a 30-kDa cutoff. The following samples were analyzed: unconcentrated supernatant (UNCS), concentrated (CS), and flow-through (FT). NC: negative control—supernatant collected from nontransfected COS-1cells. The antigens were analyzed through Western blotting performed using rabbit anti-SFTSV Gc polyclonal antibodies (**A**), rabbit anti-SFTSV Gn polyclonal antibodies (**B**), and human anti-SFTSV Gn monoclonal antibodies (MAb4-5) (**C**). The amount of protein loaded and the original results of western blotting are presented in Fig. S4

To optimize the expression and characterization of the secreted Gn and Gc antigens, we incubated COS-1 cells under the optimal incubation conditions (32 °C for 5 days). The secreted Gn/Gc proteins were concentrated using a cellulose membrane (Fig. 3). The concentrated supernatant exhibited strong bands corresponding to the expected sizes of Gn and Gc antigens (Fig. 3A and B). In addition, potential heterodimers of Gn and Gc antigens were found near the 130-kDa marker (Fig. 3A). The human MAb4-5 mAb successfully bound to Gn antigens and Gn/Gc heterodimers in the concentrated supernatant (Fig. 3C).

**Fig. 4:**
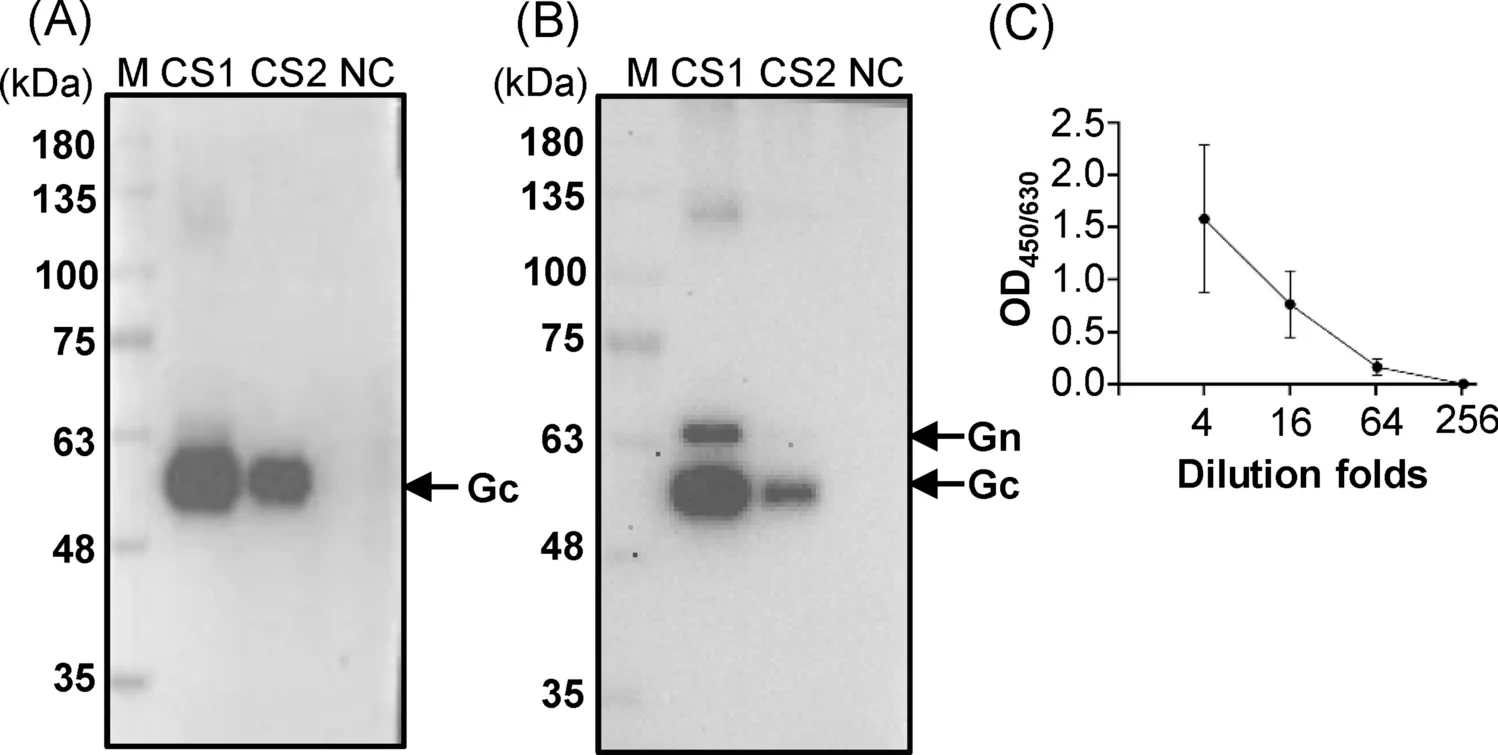
Detection of secreted and concentrated SFTSV Gn/Gc antigens through ELISA. Rabbit (**A**) and mouse (**B**) anti-SFTSV Gn/Gc polyclonal antibodies were generated by immunizing rabbit and mice with pcDNA3.1-SFTSV GB, respectively. Western blotting revealed that these antibodies recognized SFTSV Gn/Gc antigens. M: Protein ladder; CS1 and CS2: tenfold and 100-fold dilution of the concentrated Gn/Gc supernatant; NC: negative control—supernatant collected from nontransfected COS-1 cells. The amount of protein loaded and the original results of Western blotting are presented in Fig. S5. Concentrated Gn/Gc antigens (**C**) were detected through antigen-capture ELISA performed using human anti-SFTSV Gn mAb and rabbit anti-SFTSV Gn/Gc polyclonal antibodies. All experiments were performed in duplicate

To investigate whether the secreted Gn/Gc antigens could be recognized in their native form, we performed antigen-capture ELISA with the human MAb4-5 mAb. Rabbit anti-SFTSV Gn and Gc polyclonal antibodies used in Western blotting failed to recognize the secreted antigens in the ELISA test. Thus, new anti-SFTSV Gn/Gc polyclonal antibodies were generated by immunizing rabbits and mice with the pcDNA3.1-SFTSV GB plasmids. Rabbit anti-Gn/Gc polyclonal antibodies recognized Gc antigens, but the response to Gn antigens remained unclear (Fig. 4A). By contrast, mouse anti-SFTSV Gn/Gc polyclonal antibodies recognized both Gn and Gc antigens (Fig. 4B). Among the evaluated ELISA configurations, the most effective format involved coating with MAb4-5 mAbs and detecting with rabbit anti-Gn/Gc polyclonal antibodies, which enabled recognition of secreted Gn/Gc antigens in their native forms (Fig. 4C). Therefore, the secreted SFTSV Gn/Gc antigens retained the MAb4-5 epitope. These findings highlight the potential of SFTSV Gn/Gc antigens for detecting antibodies in virus-infected hosts.

### Gn/Gc antigens assemble into VLPs

**Fig. 5:**
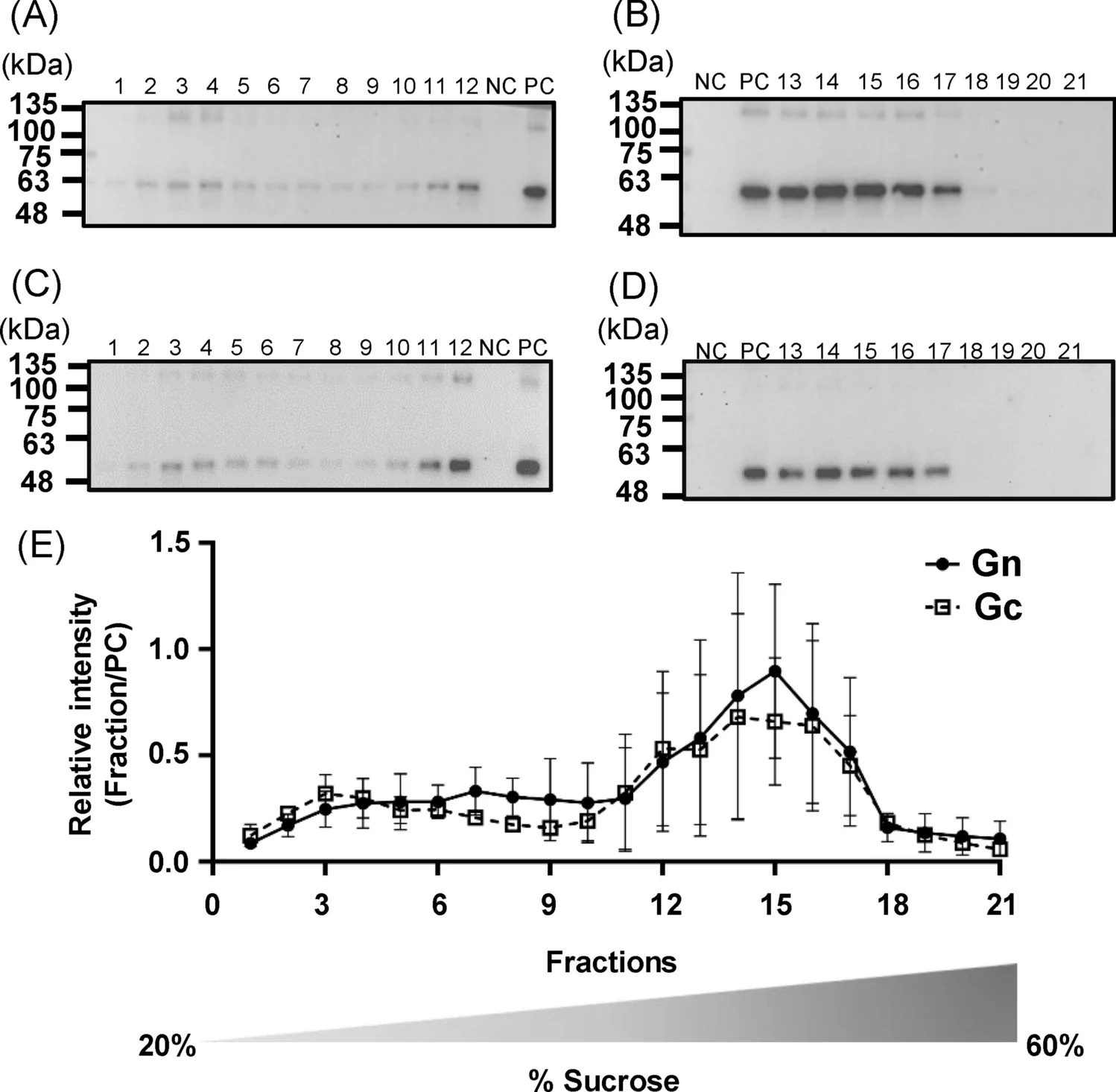
Distribution of SFTSV Gn/Gc antigens along a sucrose density gradient. Concentrated SFTSV Gn/Gc supernatant was subjected to a 20 to 60% sucrose density gradient centrifugation. Gn and Gc antigens in fractions collected from the top (fraction 1; 20% sucrose) to the bottom (fraction 21; 60% sucrose) were analyzed through western blotting performed using rabbit anti-SFTSV Gn polyclonal antibodies (**A** and **B**) and rabbit anti-SFTSV Gc polyclonal antibodies (**C** and **D**). M: protein ladder; NC: negative control—supernatant collected from non-transfected COS-1 cells; PC: positive control—concentrated SFTSV Gn/Gc supernatant. Intensity of Gn or Gc (**E**) for each fraction was plotted against that of PC. All experiments were performed in duplicate. The amount of protein loaded and the original results of Western blotting are presented in Fig. S6

Western blotting revealed that Gn and Gc proteins interact to form heterodimers (Fig. 3), raising the question of whether expressing the complete glycoprotein is sufficient to drive the formation of SFTSV VLPs. To investigate this, we concentrated the supernatants collected from COS-1 cells transfected with the pcDNA3.1-SFTSV GB plasmid and subjected it to a 20–60% sucrose density gradient centrifugation (Fig. 5). A total of 21 fractions were collected from each gradient and stained with rabbit polyclonal antibodies against Gn (Fig. 5A and B) or Gc (Fig. 5C and D). Gn and Gc yields were found to increase as the sucrose density increased from fractions 11 to 17. In fractions 18–21, both antigens were virtually undetectable.

**Fig. 6:**
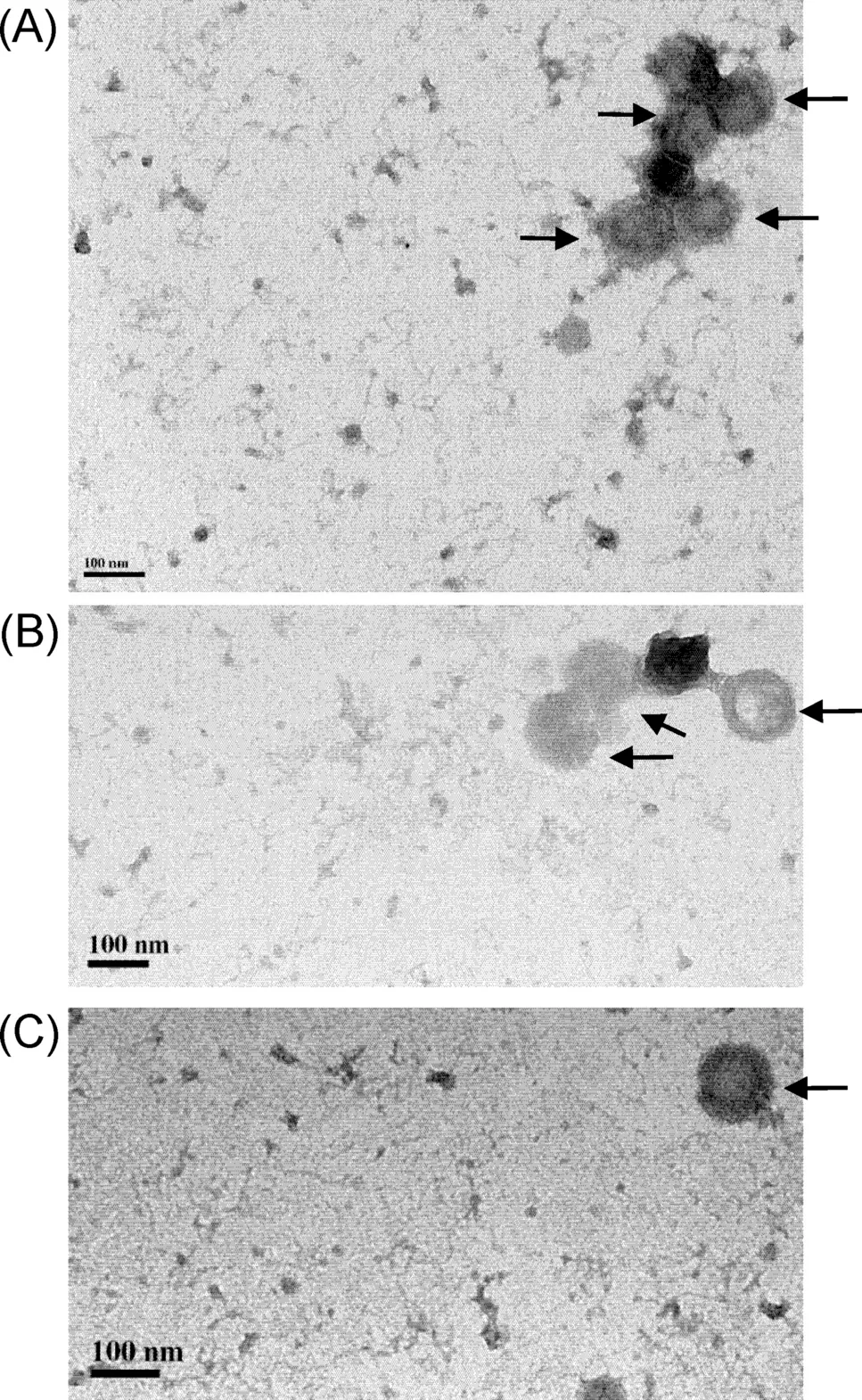
Transmission electron microscopy observation of SFTSV Gn/Gc antigens. **A** to **C** Fractions 13–17 of concentrated Gn/Gc antigens were pooled, stained with 2% uranyl acetate, and observed under a transmission electron microscope. SFTSV virus-like particles are indicated by arrows. The scale bar represents a diameter of 100 nm

Quantification of the intensity of Gn or Gc against that of the positive control (Fig. 5E) revealed co-peaking in the same fractions, suggesting that these antigens interact or form complexes with similar densities. Higher antigen levels were detected in fractions with higher sucrose concentrations, indicating the formation of larger assemblies. To confirm the presence of SFTSV VLPs, we performed TEM on fractions 13–17, for which the highest antigen levels of Gn/Gc antigens were detected. TEM revealed particles approximately 100–110 nm in size (Fig. 6). These findings indicate that the intracellular expression of the complete SFTSV glycoprotein leads to the formation and secretion of VLPs.

## Discussion

Mammalian cells were found to remain viable at temperatures below 37 °C, although their growth rates were lower under these conditions. Cultivating mammalian cells at lower temperature enhances the yield of recombinant proteins by upregulating transcription, stabilizing mRNA, and promoting posttranslational modifications (Liu and Naismith 2008; Yoon et al. 2003). However, the extent of this temperature-dependent enhancement depends on cell type and protein of interest (Esposito et al. 2020; Lin et al. 2015). The yields of SFTSV Gn/Gc antigens and VLPs were higher in HEK293T and COS-1 cells cultivated at 32 °C than in those cultivated at 37 °C or 28 °C. Notably, transfected HEK293T cells incubated at 28 °C exhibited a tendency to detach from their culture vessels, possibly indicating low cell viability at this temperature. We previously demonstrated improved secretion of flavivirus VLPs in COS-1, Vero, CHO-K1, and HEK293T cells when transfected cells were shifted from 37 to 28 °C 6 h after transfection (Fan et al. 2024). In the present study, for both HEK293T and COS-1 cells, the highest levels of Gc antigens were detected 5 DPI when the incubation temperature was 32 °C (Fig. 2). Gc antigen levels declined markedly after this time point. This finding suggests the instability of SFTSV Gc antigens or Gn/Gc VLPs at 32 °C.

The recently published Cryo-EM structure of SFTSV reveals viral particles with a diameter of approximately 110 nm organized in icosahedral symmetry. On these particles, ectodomains of Gn and Gc assemble into heterodimers, which further interact as groups of five or six heterodimers (Du et al. 2023). We detected Gn/Gc heterodimers in the secreted SFTSV antigens (Fig. 3), which suggested that Gn/Gc antigens interacted in a manner resembling the interaction of native viral particles. Sucrose density gradient centrifugation revealed that Gn/Gc antigens clustered in fractions with higher sucrose concentrations. This finding prompted us to investigate the potential of these antigens for VLP formation.

In members of *Phenuiviridae*, VLP formation based solely on glycoprotein expression has varying degrees of success. For example, Mandell et al. (2010) reported the secretion of Rift Valley fever virus (RVFV) VLPs solely through Gn/Gc expression, whereas Piper et al. (2011) reported that genomic RNA and the N protein are needed for the efficient release of RVFV VLPs from transfected cells. However, Överby et al. (2006) prepared Uukuniemi virus VLPs through Gn/Gc expression alone. Brennan et al. (2015) developed an infectious M-segment–based minigenome system for SFTSV; this system enables viral transcription and replication in T7-polymerase-expressing cells through co-transfection of plasmids carrying full-length cDNA clones of the L segment and N protein. Using these cDNA clones, Brennan et al. (2015) generated recombinant SFTSV with characteristics similar to those of the wild-type virus. The minigenome system is useful for screening antiviral drugs and studying the effects of mutations on SFTSV replication. However, it carries infection risks and requires BSL 2 + or BSL 3 containment. By contrast, our VLPs contain no genetic material and are therefore noninfectious; thus, they can be handled in low-BSL facilities. In this study, TEM confirmed the production of SFTSV VLPs, revealing particles approximately 100 nm in diameter, consistent with the reported morphology of SFTSV (Du et al. 2023). In our preliminary experiments, SFTSV VLPs recognized by MAb4-5 mAb (Fig. 4C) could detect antibody responses in a documented case of SFTS in Taiwan (unpublished data). This finding highlights the potential of SFTSV VLPs for serological surveillance and diagnostics.

The formation of SFTSV VLPs presents exciting opportunities for vaccine development. Various SFTSV vaccine candidates have been explored thus far—for example, live-attenuated, DNA, whole-inactivated viruses, viral vectors, protein subunit, and mRNA vaccines (Kim et al. 2024). However, live-attenuated viruses, particularly segmented genomes viruses (e.g., Bunyaviruses), may mutate and thereby revert to a virulent form (Noad and Roy 2003; Tariq et al. 2021). This possibility is concerning given the high case-fatality rate of SFTS in older adults, an immunocompromised population.

Other vaccine types have their own disadvantages. DNA vaccines may integrate into the host genome, whole-inactivated vaccines require large BSL-3 facilities, viral vector vaccines may be neutralized by pre-existing immunity, and protein subunit vaccines may exhibit improper folding, resulting in suboptimal immunogenicity (Noad and Roy 2003). Kim et al. (2024) developed a lipid nanoparticle-encapsulated mRNA vaccine on the basis of the SFTSV Gn head domain. Although this vaccine exhibited promising results in mice, it did not leverage the potential benefits of anti-Gc antibodies (Sano et al. 2024). In fact, some anti-Gc monoclonal antibodies induced in mice exhibited synergistic neutralizing effects when combined with the anti-Gn MAb4-5 mAb (Sano et al. 2024).

Self-assembling SFTSV Gn/Gc VLPs could help overcome the limitations of other vaccine candidates. VLPs are noninfectious, can be cultured under less stringent biosafety conditions, and benefit from mammalian expression systems, which enable posttranslational modifications resembling those of real viruses (Nooraei et al. 2021). VLP-based vaccines against pathogens such as hepatitis B virus, human papillomavirus, hepatitis E virus, and malaria have been demonstrated to elicit robust antibody and cell-mediated immune responses (Tariq et al. 2021). Mandell et al. (2010) proposed a chimeric RVFV VLP comprising RVFV Gn/Gc, RVFV N protein, and Moloney murine leukemia virus gag protein as a vaccine candidate; this vaccine was found to protect mice from RVFV challenge.

We demonstrated that the complete SFTSV glycoprotein can self-assemble into SFTSV VLPs without requiring any other viral proteins or RNA. Furthermore, low-temperature cultivation enhances the yield of SFTSV VLPs. Currently, we are devising a strategy for optimizing the production of SFTSV VLPs by using alternative transfection reagents (e.g., polyethylenimine) and suspended HEK293 cells. Overall, our findings highlight the potential of SFTSV VLPs for detecting antibody responses in SFTS patients. Furthermore, these VLPs hold promise for vaccine development in the future.

Supplementary information.

## Supplementary Information

Below is the link to the electronic supplementary material.

Supplementary file1 (PDF 3270 KB)

## Data Availability

Data supporting the findings of this study are available from the corresponding author upon reasonable request.
