# Supplementary material for: Low-temperature enhances production of severe fever with thrombocytopenia syndrome virus virus-like particles
Source: Appl Microbiol Biotechnol. 2025 Mar 3;109:56. doi: 10.1007/s00253-025-13436-y (PMC11876217; doi:10.1007/s00253-025-13436-y)
Supplement: Supplementary file 1 [file 253_2025_13436_MOESM1_ESM.pdf]

Applied Microbiology and Biotechnology
Supplementary file

Low-Temperature enhances production of Severe Fever with Thrombocytopenia Syndrome Virus Virus-Like Particles

Isabelle Loop[a, 1], Yuan-Dun Ke[a, 1], Wei-June Chen[b], Kun-Hsien Tsai[c, d], Wei-Li Hsu[e], Yi-Chin Fan[a, f, *]

[a] Institute of Epidemiology and Preventive Medicine, College of Public Health, National Taiwan University, Taipei, Taiwan
[b] Department of Public Health and Parasitology, Chang Gung University, Taoyuan, Taiwan
[c] Institute of Environmental and Occupational Health Sciences, College of Public Health, National Taiwan University, Taipei, Taiwan
[d] Global Health Program, College of Public Health, National Taiwan University, Taipei, Taiwan
[e] Graduate Institute of Microbiology and Public Health, College of Veterinary Medicine, National Chung Hsing University, Taichung, Taiwan
[f] Master of Public Health Degree Program, College of Public Health, National Taiwan University, Taipei, Taiwan

* Corresponding author: Yi-Chin Fan. Institute of Epidemiology and Preventive Medicine, College of Public Health, National Taiwan University, Taipei, Taiwan.

E-mail address: yichinfan@ntu.edu.tw (YC. Fan)

[1] These authors have contributed equally.

Sequence:
ATGATGAAGGTGATCTGGTTTAGCTCCCTGATCTGCCTGGTGATCCAGTGTT
CCGGCGATACCTCCCCTATCATCTGTGCCGGCCCCATCCACAGCAATAAGAG
CGCCAACATCCCTCACCTGCTGGGCTACAGCGAGAAGATCTGCCAGATCGA
TAGACTGATCCACGTGAGCAGCTGGCTGAGGAATCACTCCCAGTTCCAGG
GCTACGTGGGCCAGAGAGGCGGCAGATCCCAGGTGAGCTACTTCCCTGCC
GAGAATAGCTACAGCAGATGGTCCGGCCTGCTGAGCCCCTGTGACGCCGAT
TGGCTGGGCATGCTGGTGGTGAAGAAGGCCAAGGGCAGCGATATGATCGT
GCCCGGCCCCTCCTACAAGGGCAAGGTGTTCTTTGAGAGGCCTACATTCGA
CGGCTACGTGGGATGGGGCTGCGGCAGCGGAAAGAGCAGGACAGAGAGC
GGCGAGCTGTGTAGCAGCGATAGCGGCACATCCAGCGGCCTGCTGCCCAG
CGACAGAGTGCTGTGGATCGGCGACGTGGCCTGCCAGCCCATGACCCCAA
TCCCCGAGGAGACATTTCTGGAGCTGAAGTCCTTCAGCCAGAGCGAGTTT
CCTGATATCTGTAAGATCGACGGCATCGTGTTCAACCAGTGCGAGAGCGAG
TCCCTGCCTCAGCCCCTGGACGTGGCCTGGATGGATGTGGGCCACTCCCAC
AAGATCATCATGAGAGAGCACAAGACAAAGTGGGTGCAGGAGAGCTCCTC
CAAGGACTTTGTGTGTTACAAGGAGGGCACAGGCCCCTGTAGCGAGTCCG
AGGAGAAGACATGTAAGACATCCGGCAGCTGCAGAGGCGATATGCAGTTT
TGTAAGGTGGCCGGCTGTGAGCACGGCGAGGAGGCTTCCGAGGCCAAGTG
TAGGTGTAGCCTGGTGCACAAGCCTGGCGAGGTGGTGGTGTCCTACGGCG
GCATGAGAGTGAGGCCTAAGTGTTACGGCTTTTCCAGAATGATGGCCACCC
TGGAGGTGAACCCCCCCGAGCAGAGAGTGGGCCAGTGCACAGGCTGCCA
CCTGGAGTGTATCAACGGCGGCGTGAGACTGATCACCCTGACAAGCGAGC
TGAAGAGCGCCACCGTGTGCGCCAGCCACTTTTGCTCCTCCGCCACCTCCG
GCAAGAAGAGCACAGAGATCCAGTTTCACAGCGGCTCCCTGGTGGGCAAG
GCCGCTATCCACGTGAAGGGCGCCCTGGTGGACGGCACCGAGTTCACATTT
GAGGGCTCCTGCATGTTCCCTGATGGCTGCGATGCCGTGGATTGTACATTTT
GCAGAGAGTTCCTGAAGAATCCCCAGTGCTACCCTGCCAAGAAGTGGCTG
TTCATCATCATCGTGATCCTGCTGGGCTATGCCGGCCTGATGCTGCTGACAA
ACGTGCTGAAGGCCATCGGCGTGTGGGGCTCCTGGGTGATCGCCCCTGTG
AAGCTGATGTTCGCCATCATCAAGAAGCTGATGAGGAGCGTGAGCTGCCTG
ATGGGCAAGCTGATGGATAGGGGCAGACAGGTGATCCACGAGGAGATCGG
CGAGAATAGGGAGGGCAATCAGGATGACGTGAGAATCGAGATGGCCAGGC
CCAGAAGGGTGAGACACTGGATGTACAGCCCCGTGATCCTGACCATCCTGG
CCATCGGCCTGGCCGAGGGCTGCGATGAGATGGTGCACGCCGACTCCAAG
CTGGTGAGCTGTAAGCAGGGCGGCGGCAACATGAAGGAGTGCGTGACCAC
AGGCAGGGCCCTGCTGCCCGCTGTGAATCCTGGCCAGGAGGCCTGCCTGC
ACTTTACAGCCCCTGGCTCCCCTGACAGCAAGTGCCTGAAGATCAAGGTG
AAGAGAATCAACCTGAAGTGTAAGAAGAGCTCCTCCTACTTTGTGCCTGAC
GCCAGGAGCAGATGTACATCCGTGAGGAGGTGCAGATGGGCCGGCGACTG
CCAGTCCGGCTGCCCTTCTCACTTCACATCCAATTCCTTCTCCGATGATTGG
GCCGGCAAGATGGACAGGGCCGGCCTGGGATTCTCCGGCTGTTCCGATGG
CTGCGGCGGCGCTGCTTGCGGATGTTTCAACGCCGCCCCCAGCTGTATCTT
CTGGAGGAAGTGGGTGGAGAACCCTCACGGCATCATCTGGAAGGTGAGCC
CTTGTGCCGCCTGGGTGCCCAGCGCTGTGATCGAGCTGACCATGCCTTCCG
GCGAGGTGAGGACATTCCACCCTATGAGCGGCATCCCCACCCAGGTGTTTA
AGGGCGTGTCCGTGACCTACCTGGGCAGCGATATGGAGGTGAGCGGCCTG
ACCGATCTGTGTGAGATCGAGGAGCTGAAGTCTAAGAAGCTGGCCCTGGC
CCCTTGTAACCAGGCCGGCATGGGCGTGGTGGGCAAGGTGGGAGAGATCC
AGTGCTCCTCCGAGGAGTCCGCCAGAACAATCAAGAAGGACGGCTGTATC

TGGAATGCCGACCTGGTGGGCATCGAGCTGAGAGTGGACGACGCCGTGTG
CTACAGCAAGATCACAAGCGTGGAGGCCGTGGCCAACTACTCCGCCATCC
CTACCACAATCGGCGGCCTGAGATTTGAGAGGAGCCACGACTCCCAGGGC
AAGATCTCCGGCTCCCCTCTGGACATCACAGCCATCAGGGGCTCCTTCAGC
GTGAACTACAGAGGCCTGAGGCTGTCCCTGTCCGAGATCACCGCCACATGT
ACAGGCGAGGTGACAAATGTGTCCGGCTGCTACTCCTGTATGACAGGCGCC
AAGGTGTCCATCAAGCTGCACTCCAGCAAGAACAGCACAGCCCACGTGAG
GTGCAAGGGCGACGAGACCGCCTTCTCCGTGCTGGAGGGCGTGCACAGCT
ACACAGTGTCCCTGTCCTTTGATCACGCCGTGGTGGACGAGCAGTGCCAG
CTGAACTGCGGCGGCCACGAGAGCCAGGTGACCCTGAAGGGCAATCTGAT
CTTTCTGGATGTGCCCAAGTTTGTGGATGGCAGCTACATGCAGACCTACCA
CTCCAGCGTGCCCACAGGCGCCAATATCCCTTCCCCTACAGATTGGCTGAA
CGCCCTGTTCGGCAACGGCCTGTCCAGGTGGATCCTGGGCGTGATCGGCGT
GCTGCTGGGCGGACTGGCCCTGTTCTTCCTGATCATGTCCCTGTTCAAGCT
GGGCACAAAGCAGGTGTTTAGGAGCAGAGCCAAGCTGGCC

Fig. S1 The codon-optimized sequence of Severe fever with thrombocytopenia syndrome virus (SFTSV) glycoprotein based on human-derived cells. A total of 3219 nucleotide sequences in length.

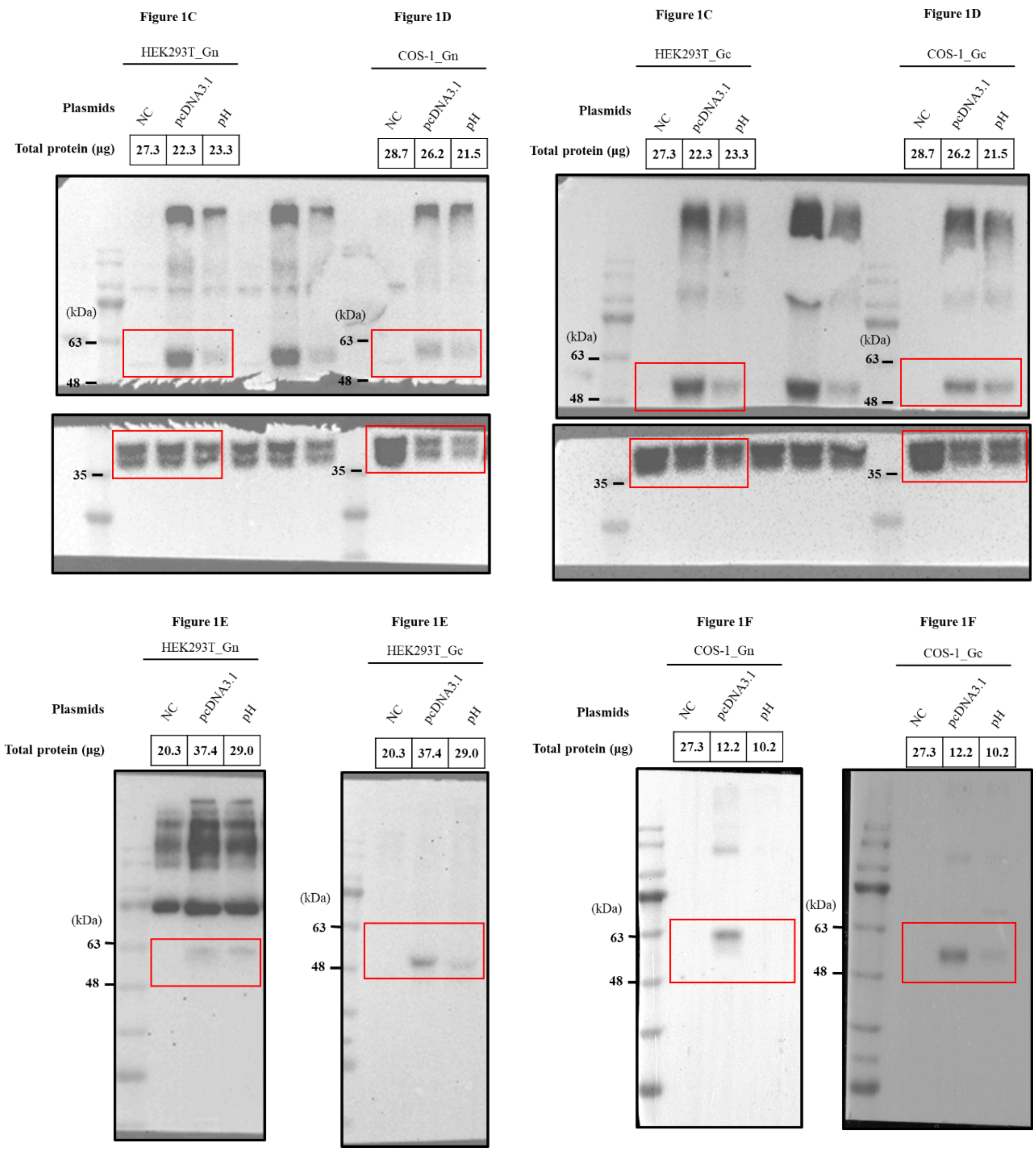

Fig. S2 The original Western blotting of Fig. 1. Sections delineated by red rectangular boxes were displayed in the Fig. 1 of the manuscript. The total protein amount (μg) used in each assay was indicated below the corresponding sample. Protein concentrations were quantified using the Thermo Scientific NanoDrop One, with bovine serum albumin (BSA) serving as the reference standard.

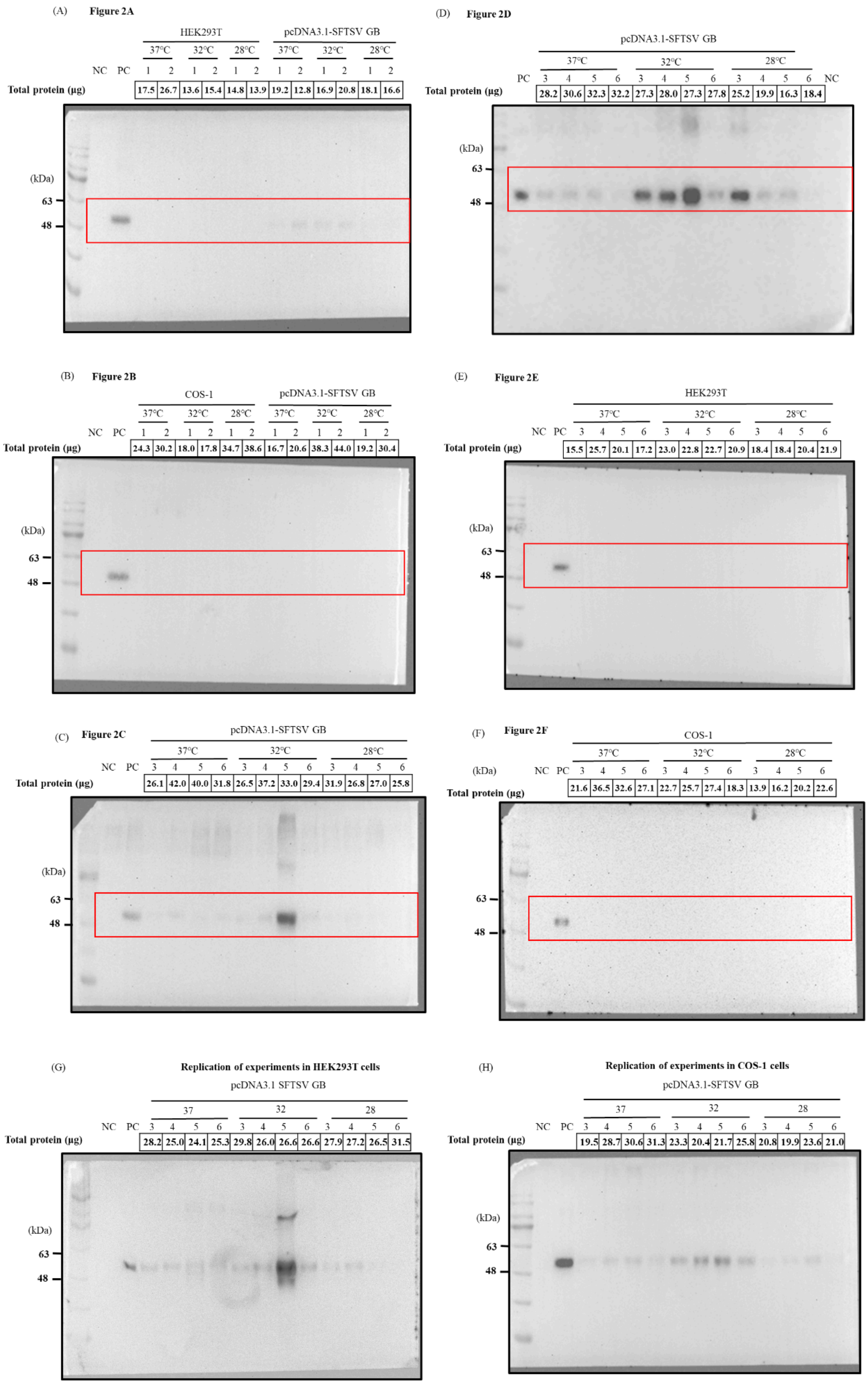

Fig. S3 The original Western blotting of Fig. 2 and replication of experiments. Sections delineated by red rectangular boxes are shown in Fig. 2 (Panels A–F) of the manuscript. Replicated experiments in HEK293T and COS-1 cells are presented in

panels G and H, respectively. The total protein amount (μg) used in each assay is indicated below each corresponding sample. Protein concentrations were quantified using the Thermo Scientific NanoDrop One, with BSA serving as the reference standard.

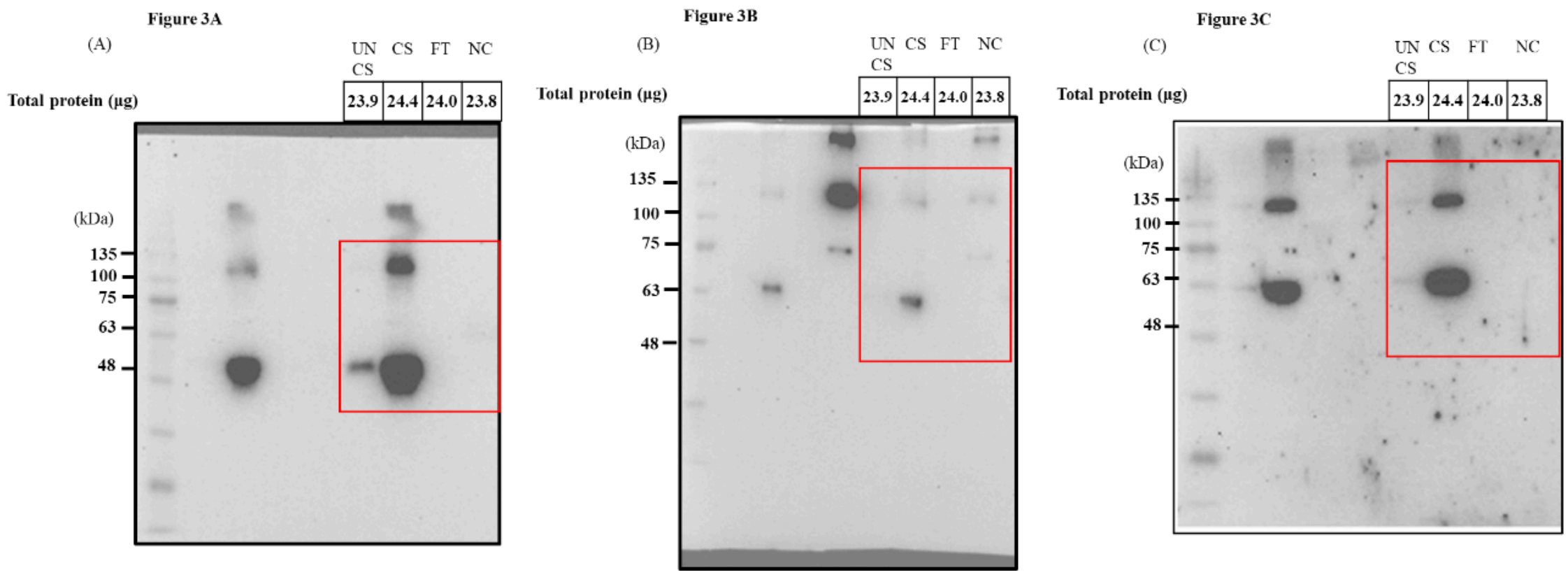

Fig. S4 The original Western blotting of Fig 3. Sections delineated by red rectangular boxes were displayed in the Fig. 3 of the manuscript. The total protein amount (µg) used in each assay was indicated below the corresponding sample. Protein concentrations were quantified using the Thermo Scientific NanoDrop One, with bovine serum albumin (BSA) serving as the reference standard.

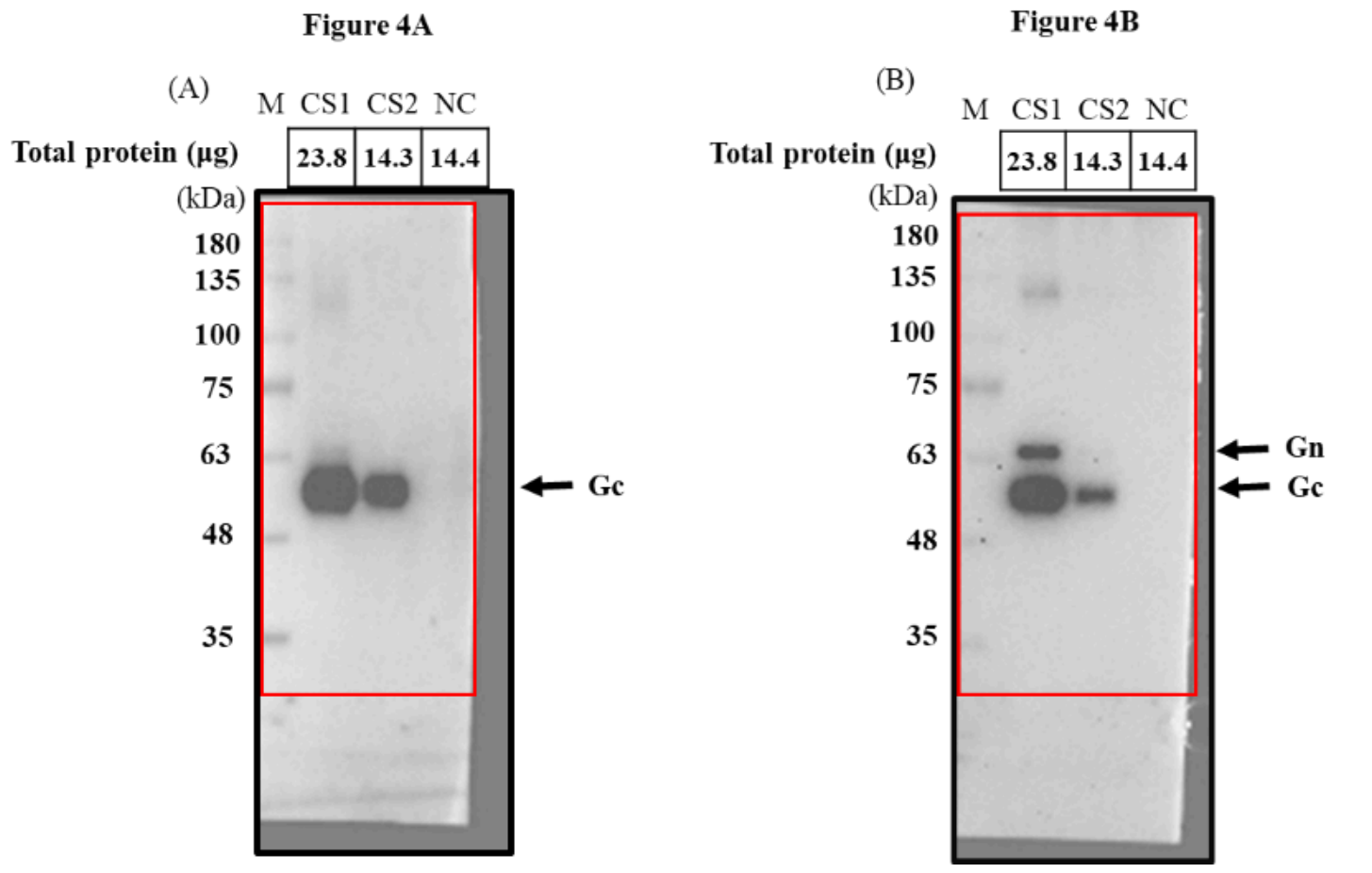

Fig. S5 The original Western blotting of Fig 4. Sections delineated by red rectangular boxes were displayed in the Fig. 4 of the manuscript. The total protein amount (μg) used in each assay was indicated below the corresponding sample. Protein concentrations were quantified using the Thermo Scientific NanoDrop One, with BSA serving as the reference standard.

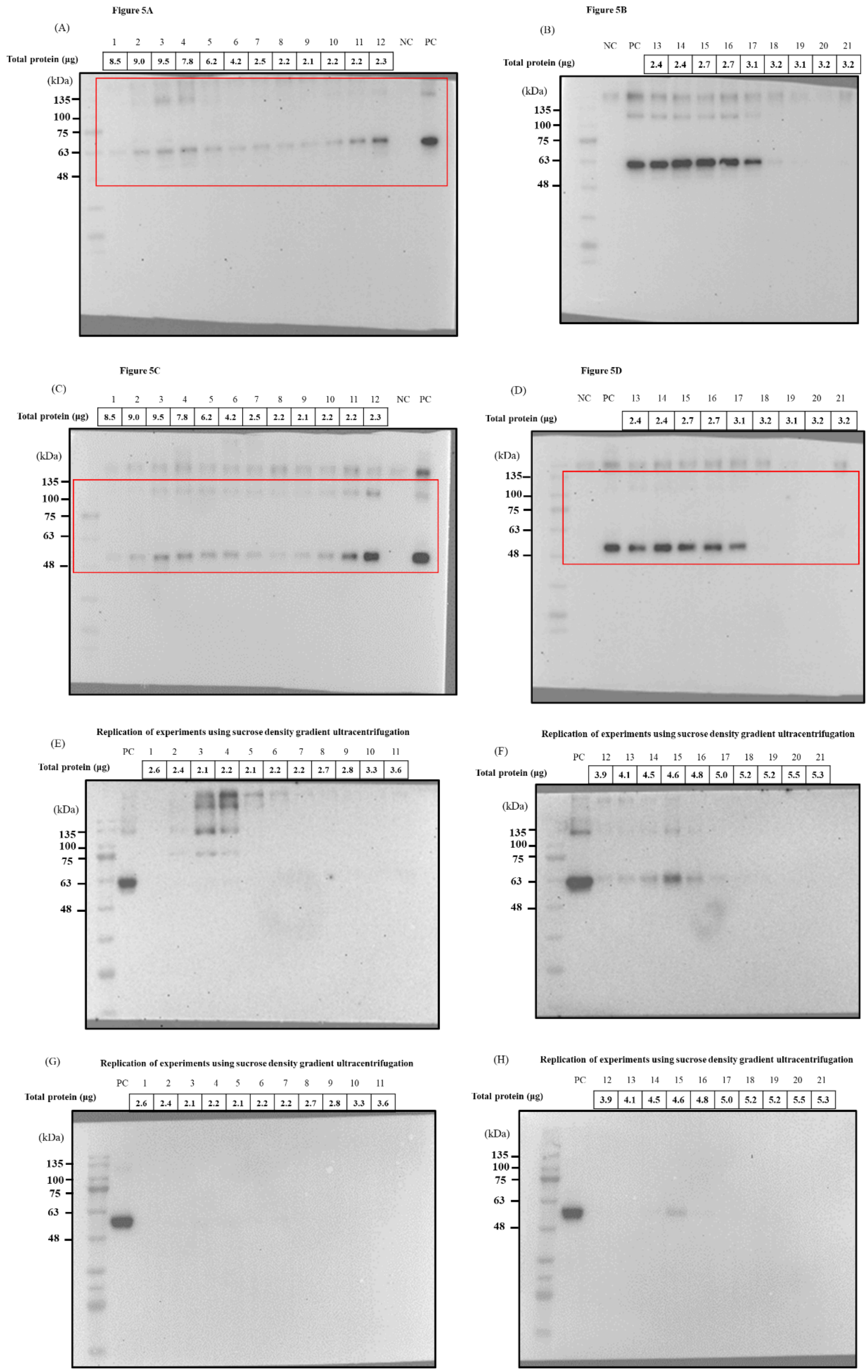

Fig S6. The original Western blotting of Fig 5 and replication of experiments. Sections delineated by red rectangular boxes are shown in Fig. 5 (Panels A–D) of the manuscript. Replicated experiments staining for Gn and Gc proteins are presented in

panels E-F and G-H, respectively. The total protein amount (μg) used in each assay is indicated below each corresponding sample. Protein concentrations were quantified using the Thermo Scientific NanoDrop One, with BSA serving as the reference standard.
